# Experimental Tests and Numerical Analysis of Aerodynamic Properties of the Composite-Made Jet-Propelled Aerial Target

**DOI:** 10.3390/ma17143575

**Published:** 2024-07-19

**Authors:** Michał Frant, Łukasz Kiszkowiak, Maciej Majcher, Piotr Zalewski

**Affiliations:** Faculty of Mechatronics, Armament and Aerospace, Military University of Technology, Kaliskiego 2 St., 00-908 Warsaw, Poland; michal.frant@wat.edu.pl (M.F.); lukasz.kiszkowiak@wat.edu.pl (Ł.K.); maciej.majcher@wat.edu.pl (M.M.)

**Keywords:** aerospace engineering, composite materials, aerial target design, aerodynamics, computational fluid dynamics CFD, wind tunnel tests

## Abstract

The design of an aircraft’s internal structure, and therefore the appropriate choice of material type, is a direct function of the performed tasks and the magnitude and type of the acting loads. The design of a durable aircraft structure with appropriate stiffness and lightness requires knowledge of the loads that will be applied to the structure. Therefore, this paper presents the results of an aerodynamic experimental test and numerical analysis of a newly designed jet-propelled aerial target. The experimental tests were carried out in a low-speed wind tunnel for a wide range of angles of attack and sideslips. Moreover, they were performed for various configurations of the airplane model. In addition, the results of the experimental test were supplemented with the results of the numerical analysis performed using computational fluid dynamics methods. During numerical analysis, specialized software based on solving partial differential equations using the Finite Volumes Method was used. This article presents the methodology of the conducted research. The results of the aerodynamic analysis are presented in the form of diagrams showing the aerodynamic force and moment components as a function of the angle of attack and sideslip. In addition, qualitative results of the flow around the plane have been presented. The results obtained prove that the adopted methods are sufficient to solve these types of problem. The aerial system was positively verified during the qualification tests of the system at the Polish Air Force training range and finally received the type certificate.

## 1. Introduction

In 1951, the Rayan Aeronautical Company designed the Firebee, the first turbojet powered air target [1], which is used successfully to this day, and soon other designs of this type appeared on the market [2,3,4,5,6]. Today, the American company Kratos is a leading provider of state-of-the-art, high-performance aerial target drones [4,5] the BQM 177 series. Reusable, turbojet-powered, target drones are primarily used for testing surface-to-air missile systems or practice engagements for fighters and air-to-air missiles.

In 2013, the Polish Ministry of Defence commissioned the development of a similar system in the country. The consortium, consisting of the Air Force Institute of Technology, the MSP Szender Company, the Warsaw University of Technology, and the Military University of Technology, was selected to implement the project under the name “ZOCP-JET2 Programmable Air Target”. The prototype set of five UAVs was deployed to the Polish Armed Forces in 2021 for further evaluation.

The Military University of Technology (MUT), as a member of the consortium, was responsible for determining the aerodynamic properties and characteristics of the aircraft (JET2), so that its performance met the requirements imposed by the MoD, i.e., a maximum speed of 150 m/s, a flight duration of 60 min, and an operational altitude of 5000 m.

The purpose of this article is to present the process of obtaining data on the aerodynamic characteristics of the designed aerial target, using experimental and numerical methods. This type of research and its results are necessary to assess the correctness of the implemented design of the flying object. The aerodynamic characteristics presented in this article, or, more precisely, the values of the coefficients of forces and moments, are necessary to assess the stability, controllability, and maneuverability of the flying object as well as to carry out further work on the structure. They allow for the estimation of the loads that the composite structure of the aerial target must carry. Many publications contain the results of experimental tests of the model in wind tunnels [7,8,9] and their comparison with numerical results. It concerns the study of the entire set [10] and the impact of its individual elements [11,12]. The aim of our research, apart from the need to obtain aerodynamic characteristics in a large range of attack and sideslip angles, with elevator deflections, was also to obtain data for validation of the numerical model, similarly to [13,14].

These are both static and functional tests [15]. Nowadays, models for tests in wind tunnels are made with 3D printing technology [16,17].

This paper is organized as follows: the research methods are described in Section 2, where the mathematical model, which is the basis for the parametric programming, is defined, and, in the second part of this section, the CFD and panel method settings are given; the obtained results are shown in Section 3 and concluded in Section 4.

## 2. Materials and Methods

### 2.1. Wind Tunnel Tests

**Figure 1:**
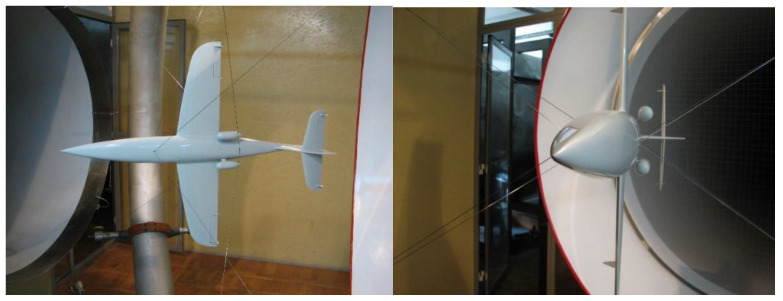
The MUT wind tunnel with the scale model of the jet aerial target.

**Figure 2:**
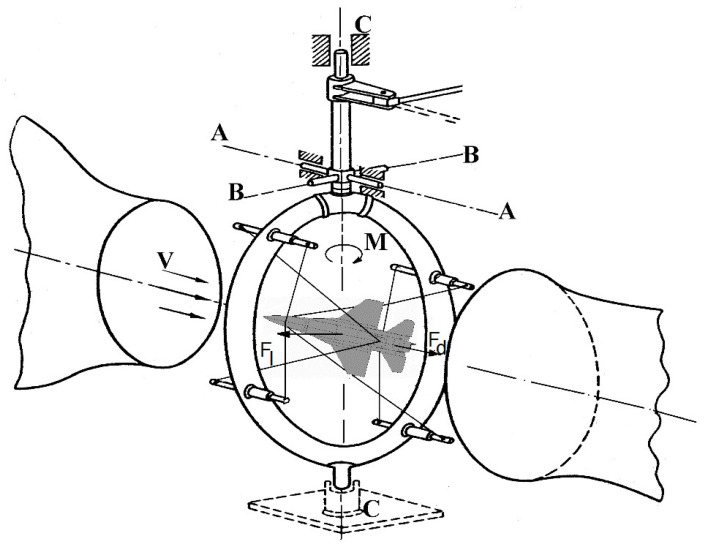
Schematic of the MUT wind tunnel measurement system (F_l_—lift force, F_d_—drag force, M—pitch moment, V—velocity vector, A—A-lift force axis, B—B-drag force axis, C—C-pitch moment axis).

The carbon fiber composite airframe of the aerial target was manufactured by the MSP InnTech Ltd. (Warsaw, Poland) [18,19] while the 1:4 scale aircraft model for wind tunnel testing was designed and “printed” using FDM (Fused Deposition Modelling) technology at the MUT. Experimental tests were carried out in a low-speed wind tunnel with a test space diameter of D = 1.1 m. The silhouette of the model under test, suspended on an annular aerodynamic balance for testing in symmetric and asymmetric flow, is shown in Figure 1, while Figure 2 shows a schematic of the measurement system in the wind tunnel. The methodology and calculation program for aerodynamic characteristics were developed based on the literature [20,21,22,23,24,25,26,27,28].

Aerodynamic tests of the aerial target model were carried out in symmetric and asymmetric flow. These tests were carried out at a velocity pressure of q = 500 [Pa] (V = 30 m/s with Reynolds number Re = 207,000 in angles of attack *α* = ±30° and sideslip angles *β* = ±30° with an increment of 2°). The aerodynamic coefficients in symmetric flow were referred to as the model wing area of S_mod_ = 0.084375 m^2^ and mean aerodynamic chord of b_A_ = 0.11475 m, and, for asymmetric flow, was referred to as the surface area of the model’s wing S_mod_ = 0.084375 m^2^ and its wingspan L_mod_ = 0.7125 m. For the model of the aircraft in a “clean” configuration (d_H_ = 0°, d_a_ = 0°, d_V_ = 0°, where d_H_ is elevator deflection, d_a_ is aileron deflection, and d_V_ is rudder deflection) tests were performed for the position of the center of the mass of the aircraft 440 mm from the nose of the fuselage. The investigated model was installed in the wind tunnel test chamber so that the axis of the pitching moment of the tunnel balance passed through the point corresponding to the center of mass of the aircraft and the longitudinal axis of the model coincided with the axis of the aircraft weight drag.

This location allowed us to determine the following:

- Drag force, i.e., the component of the resultant aerodynamic force in the direction of the longitudinal axis of the tunnel (this required the installation of an aerodynamic balance column enabling rotation about the A–A axis);
- Lift force, i.e., the component of the resultant aerodynamic force in the direction of the horizontal axis, perpendicular to the longitudinal axis of the tunnel (supporting the aerodynamic balance column allowing its rotation relative to the B–B axis);
- Pitching moment, i.e., the moment of force relative to the vertical axis of the tunnel (with the installation of an aerodynamic balance column allowing rotation relative to the C–C axis).

Aerodynamic tests of the aerial target in the wind tunnel were performed for the model in a “clean” configuration and in elevator deflection in the range d_H_ = −30° ÷ +30° with an increment of 5°. The tests were carried out in the range of angles of attack *α* = ±30° with an increment of 2° and at a dynamic pressure of q = 500 Pa, corresponding to a relative wind velocity of V ≈ 30 m/s and a Reynolds number Re ≈ 207,000. The strain gauges of the measurement system gave results with an accuracy of ±0.01 N, while the pressure transducer gave results with an accuracy of ±1 Pa. The forces acting on the object were transferred through the string to the ring of the aerodynamic balance, which, rotating around the A–A, B–B, or C–C axes, transferred the forces to the strain gauges.

### 2.2. Computational Fluid Dynamics Analysis

Dynamic development of microprocessor technology and methods of Computational Fluid Dynamics has enabled the simulation of many phenomena occurring during the flow of fluids around solid bodies. In the theory of fluid mechanics, movement of liquids and gases is described by a system of differential equations [29,30]:

- The Navier Stokes equation (equation of momentum conservation) in the following form:

(1) $$\frac{\partial }{\partial t}\left(\rho \vec{v}\right)+\nabla \cdot \left(\rho \vec{v}\vec{v}\right)=-\nabla p+\nabla \left(\overset{̿}{\tau }\right)+\rho \vec{g}+\vec{F}$$

where:

*p*—static pressure;
$\rho \vec{g}$ and $\vec{F}$ are, respectively, gravitational forces and external forces, e.g., increasing as a result of flow through a dispersed phase;
$\overset{̿}{\tau }$—stress tensor.

(2) $$\overset{̿}{\tau }=\mu \left[\left(\nabla \vec{v}+{\nabla \vec{v}}^{T}\right)-\frac{2}{3}\nabla \cdot \vec{v}I\right]$$

where:

μ—kinematic viscosity;
*I*—unit matrix.

- Equation of flow continuity (mass conservation equation in relation to fluid treated as a continuous medium) in the following form:

(3) $$\frac{\partial \rho }{\partial t}+\nabla \cdot \left(\rho \vec{v}\right)=S_{m}$$

where:

*S_m_*—mass source (e.g., as a result of evaporation of the dispersed phase).

- Energy conservation equation in the following form:

(4) $$\frac{\partial }{\partial t}\left(\rho E\right)+\frac{\partial }{\partial x_{i}}\left(u_{i}\left(\rho E+p\right)\right)=\frac{\partial }{\partial x_{j}}\left[\left(k+\frac{c_{p}\mu_{t}}{{Pr}_{t}}\right)\frac{\partial T}{\partial x_{j}}+{u_{i}\left(\tau_{ij}\right)}_{eff}\right]+S_{h}$$

where:

*k*—thermal conductivity;
*E*—total energy;
${\left(\tau_{ij}\right)}_{eff}$—shear stress tensor.

(5) $${\left(\tau_{ij}\right)}_{eff}=\mu_{eff}\left(\frac{\partial u_{j}}{\partial x_{i}}+\frac{\partial u_{i}}{\partial x_{j}}\right)-\frac{2}{3}\mu_{eff}\left(\frac{\partial u_{k}}{\partial x_{k}}\delta_{ij}\right)$$

Solving them in the general case is possible only by using numerical methods, e.g., finite volume methods. The above equations are transformed into an integral form:

(6) $$\frac{\partial }{\partial t}∭QdV+\iint FdA=0$$

in which *Q* is used to denote values that are subject to laws of conservation (of mass, momentum, energy) inside a cell, *F* is a vector of quantities characterizing the stream exchanged with the cell environment, *V* is the volume of a single control cell, and *A* is its external surface. Equations written in this way are solved using the iterative method (successive approximations). The size of cells in the domain reproducing the air area around the studied geometry is selected so as to accurately reflect the unevenness of the flow field. Unfortunately, this is a very demanding method when it comes to computing resources, both in terms of used memory and computing performance. In the case of the geometry of an entire aircraft, calculations are most often made on a computer consisting of several to several dozen parallel working units (nodes), where each analyzes a separate fragment of the computational mesh.

**Figure 3:**
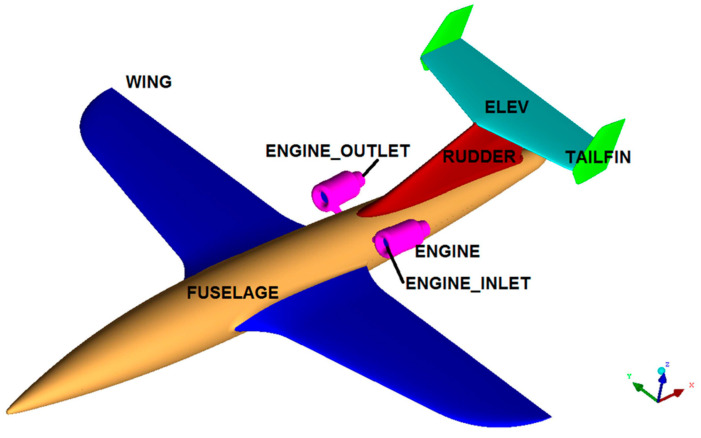
Division of the airframe surface into calculation zones.

At the stage of the evaluation of the aerial target configuration, numerical analysis was performed using Computational Fluid Dynamics methods (Figure 3). Calculations were performed using ANSYS Fluent software ver. 15 based on the finite-volume differential equation (FVM) [29,30]. The software allows for the analysis of incompressible and compressible flows, with optional consideration of flow viscosity [31,32]. When numerical aerodynamic analysis was performed in symmetrical flow, the symmetry of the flow field was assumed, and the flow was assumed to be stationary and stabilized. In numerical studies, the Spalart–Allmaras turbulence model was used, which is a typical model in the field of numerical analysis of external flows of flying objects. The ICEM CFD software ver. 15, part of the ANSYS package ver. 15, was used to generate computational meshes. This software is an advanced preprocessing tool that allows fully preparing a geometric model, i.e., building or importing geometry from CAD software Siemens NX 2020, as well as repairing and simplifying such geometry [33,34,35,36,37,38].

## 3. Results and Discussion

### 3.1. Aerodynamic Characteristics of an Aerial Target Model in Symmetric Flow

The results of the wind tunnel tests are depicted in the form of graphs showing the values of aerodynamic coefficients and tables with the most important results:

- In symmetric flow
  (a) C_D_ = f(*α*)—drag coefficient as a function of angle of attack (AOA);
  (b) *C_L_* = f(*α*)—lift force coefficient as a function of AOA;
  (c) *C_m_* = f(*α*)—pitching moment coefficient as a function of AOA;
  (d) E = L/D = f(*α*)—aerodynamic efficiency in function of AOA;
  (e) *C_L_* = f(C_D_)—lift force coefficient as a function of the drag force coefficient.
- In asymmetric flow
  (a) C_D_ = f(*β*)—drag coefficient as a function of the sideslip angle;
  (b) C_y_ = f(*β*)—lateral force coefficient as a function of the sideslip angle;
  (c) *C_n_* = f(*β*)—coefficient of yawing moment as a function of the angle of side inclination.

Symmetric flow tests were performed on a wide range of changes in elevator deflection. Knowledge of the object’s behavior at various elevator deflections is extremely important from the point of view of static longitudinal stability. This allows you to assess whether the deflection of the wheel does not cause a loss of stability. At the same time, the influence of elevator deflection on other aerodynamic characteristics was also examined.

#### 3.1.1. Aerodynamic Drag Coefficient C_D_ = f(*α*, d_H_)

**Figure 4:**
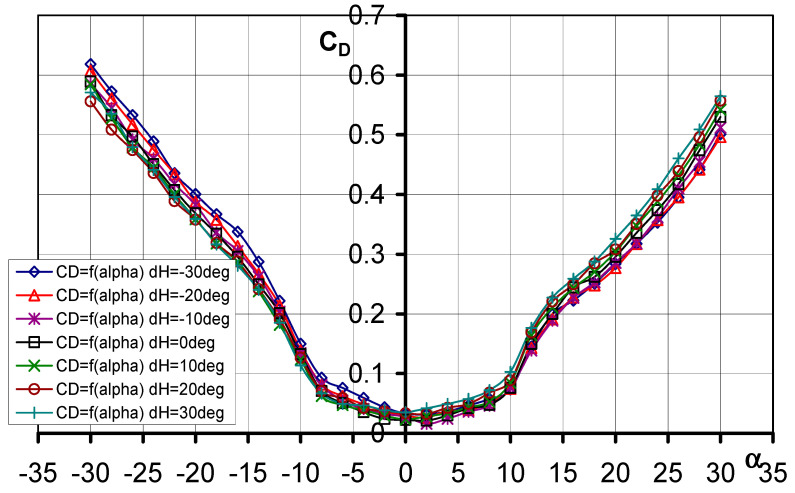
Characteristics of C_D_ = f(*α*) for different elevator defection angles d_H_.

The influence of elevator deflection on the characteristic of the drag coefficient is shown in Figure 4, while Table 1 summarizes the most important data. The curves obtained have a somewhat parabolic shape. The minimum drag coefficient values were noted for angles of attack *α* = 2° and *α* = 0°. Among all the configurations of the tested models, the lowest value of the drag coefficient was obtained for the model with elevator deflection d_H_ = −10°, and it was C_D_ = 0.01599.

#### 3.1.2. Lift Force Coefficient *C_L_* = f(*α*, d_H_)

**Figure 5:**
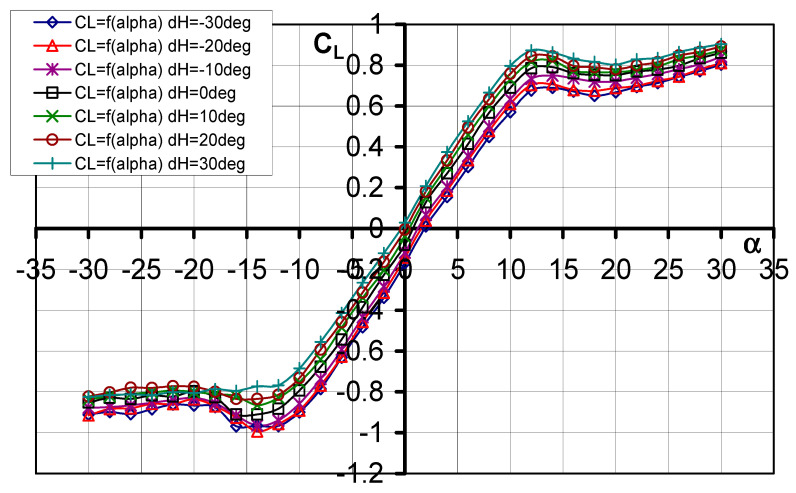
Characteristics of *C_L_* = f(*α*) for different elevator deflection angles d_H_.

The influence of elevator deflection on the characteristic of the lift force coefficient is presented in Figure 5, while Table 2 summarizes the more relevant data. Characteristic curves are linear in the range of angles of attack from *α* = −10° to *α* = 10°. It should be noted that for the studied model there is no classic sharp drop in the values of the lift force coefficient after exceeding *α*_cr_. Here, we see that after exceeding *α*_cr_ there is a gentle decrease in the value of the lift force coefficient *C_L_*, and then we observe a small continuous increase in the value of this coefficient up to *α* = 30°.

#### 3.1.3. Pitching Moment Coefficient *C_m_* = f(*α*, d_H_)

**Figure 6:**
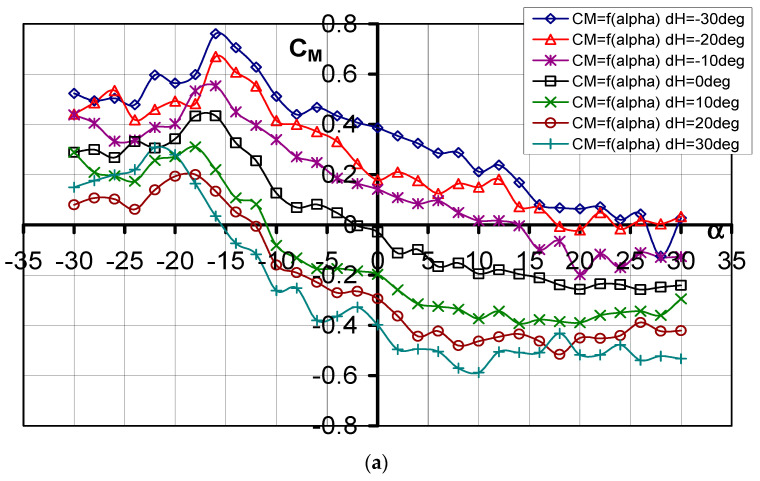

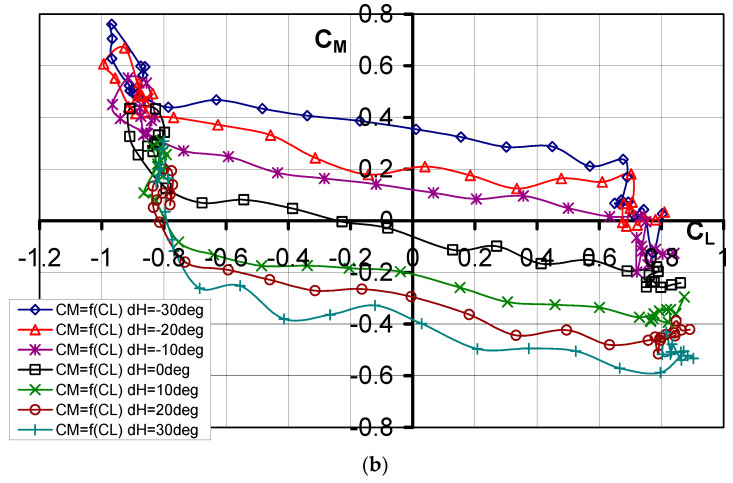
(**a**) Characteristics of *C_m_* = f(*α*) for different elevator deflection angles d_H_. (**b**) Characteristics of *C_m_* = f(*C_L_*) for different elevator deflection angles d_H_.

The curves of the pitching moment coefficient as a function of the angle of attack are shown in Figure 6a, while Figure 6b shows the pitching moment coefficient as a function of the lift force coefficient. The most important values are listed in Table 3. The characteristics obtained show that the deflection of the elevator causes the characteristic *C_m_* = f(*α*) to shift downward. In each of the cases studied, derivative 7 has a similar value.

(7) $$\frac{\partial C_{m}}{\partial \alpha }$$

which means the same value of static longitudinal stability.

The static margin can be calculated by the derivative of 8 [24]:

(8) $$\frac{\partial C_{m}}{\partial C_{L}}=\frac{x_{Q}}{b_{A}}-\frac{x_{F}}{b_{A}}$$

where:

*x_Q_* is the center of mass;
*x_F_* is the aerodynamic center;
*b_A_* is the mean aerodynamic chord.

The characteristics of the pitch moment coefficient showed the aerial target model is statically stable longitudinally when the value of the angle of attack exceeds *α* = −16°. At positive deflections of the elevator, the model starts to be statically unstable longitudinally when *α* = 12° is exceeded.

#### 3.1.4. Aerodynamic Efficiency (Lift-to-Drag Ratio) E = f(*α*, d_H_)

**Figure 7:**
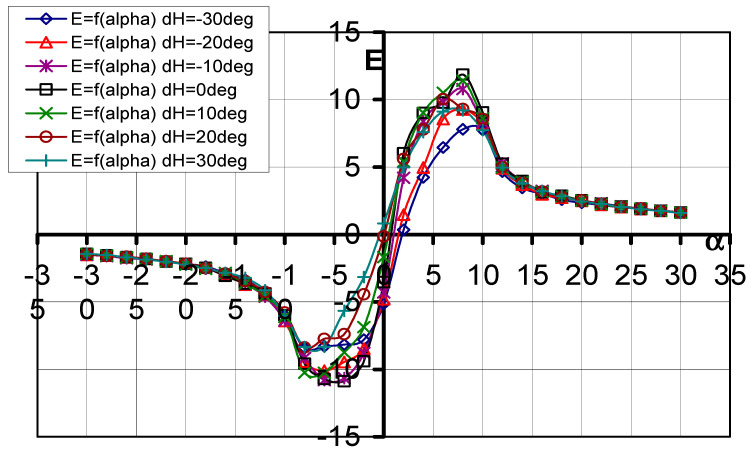
Characteristics of E = f(*α*) for different elevator deflection angles d_H_.

The aerodynamic efficiency curves as a function of the AOA E = f(*α*, d_H_) are shown in Figure 7, while, in Table 4, the numerical values at the characteristic points are shown.

As can be seen in the graphs, for all the configurations of the tested models, the optimum angle of attack is *α*_opt_ = 8°. The maximum value of aerodynamic efficiency was obtained for d_H_ = 0°.

#### 3.1.5. Lift Force Coefficient as a Function of Drag Force Coefficient *C_L_* = f(C_D_)

**Figure 8:**
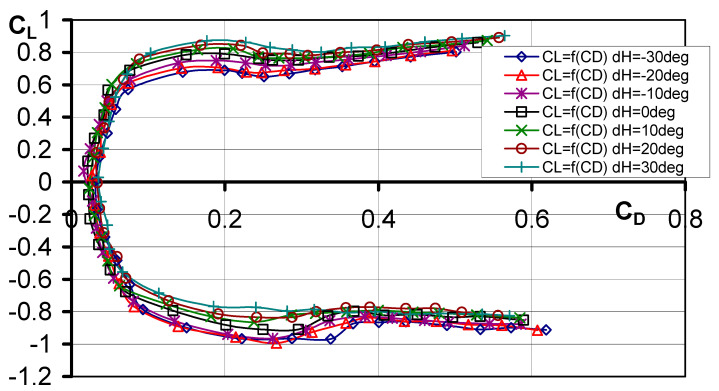
Characteristics of *C_L_* = f(C_D_) for different elevator deflection angles d_H_.

The drag polarity of the aerial target model is shown in Figure 8. Using these graphs, various aerodynamic parameters can be determined, e.g., *C*_*L* min_, *C*_*L* max_, C_Dopt_, E_max_, and E_min_, which were given with the previously analyzed characteristics.

### 3.2. Aerodynamic Characteristics of an Aerial Target Model in Asymmetric Flow

During project development, studies were also carried out on the aerodynamic characteristics of the aerial target in asymmetric flow, that is, at different angles *β*. Shown below are the characteristics obtained for the optimization of the model streamlining at an angle of attack *α* = 0°.

**Figure 9:**
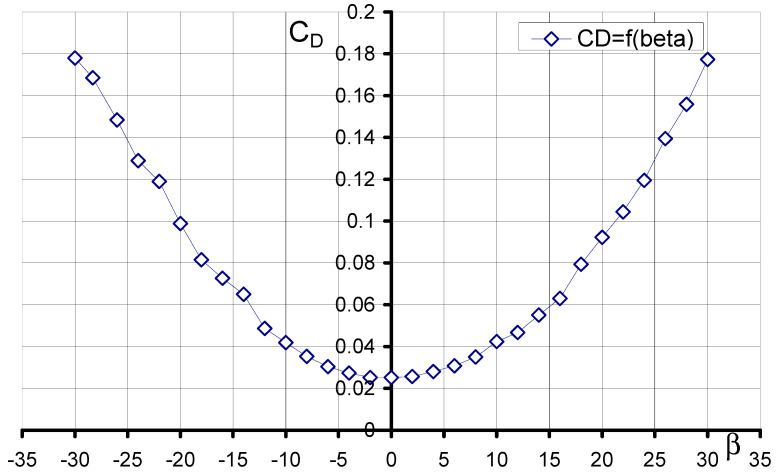
Characteristics C_D_ = f(*β*, *α* = 0°).

The characteristic of the C_D_ drag force coefficient as a function of the sideslip angle *β* is shown in Figure 9. The characteristic has a parabolic shape. It is almost symmetric about the OY axis. During the tests, the maximum value of the drag force coefficient C_D_ = 0.178 was obtained for both the sideslip angles *β* = −30° and *β* = 30°.

**Figure 10:**
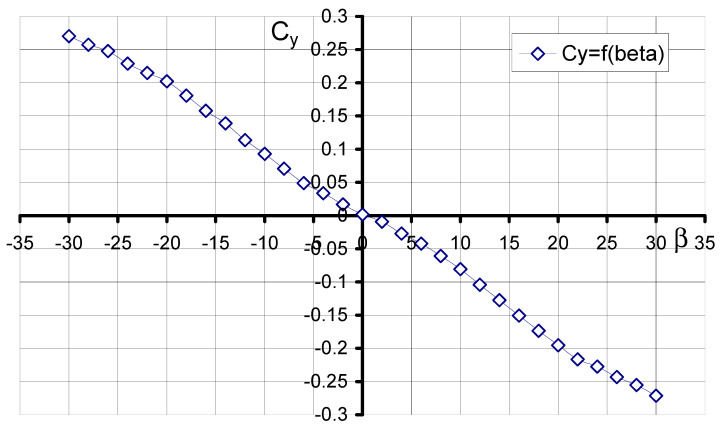
Characteristics C_y_ = f(*β*, *α* = 0°).

**Figure 11:**
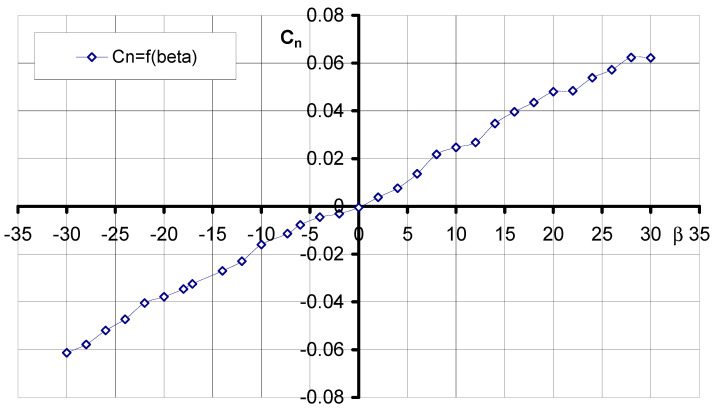
Characteristics *C_n_* = f(*β*, *α* = 0°).

Figure 10 shows the characteristic of the lateral force coefficient as a function of the angle of sideslip. The characteristic obtained has a linear shape. Maximum values were obtained for *β* = −30° as well as *β* = 30° for C_y_ = 0.27 and C_y_ = −0.27, respectively. For sideslip angle *β* = 0°, the value C_y_ = 0.0016 was obtained. The experimental results of the yawing moment coefficient *C_n_* as a function of the angle *β* are shown in Figure 11. The characteristic in the investigated range of sideslip angles has a nearly linear course. In addition, its course shows that the aerial target model is characterized by directional static stability, since the derivative of the yawing moment coefficient due to sideslip angle is positive (3). More information on the wind tunnel investigation of the air vehicle can be found [39].

(9) $$\frac{\partial C_{n}}{\partial \beta }>0$$

### 3.3. Numerical Analysis of the Aerodynamic Characteristics of an Aerial Target with and without Engine Thrust Effect

A non-structural mesh was generated in the area surrounding the aircraft airframe. The rectangular domain around the model size of 40 × 40 × 20 m represented half of the geometry. Five layers of prism cells simulating the boundary layer were generated around the walls of the aircraft. The thickness of the first mesh element (0.6 mm) corresponded to the turbulence parameter y+ in the range <30–200>, which is recommended for the Spalart–Allmaras turbulence model used. This model is adopted as a standard in the analysis of external flows, especially in the range of Reynolds numbers used in aviation [31]. The selected mesh size and turbulence model allowed for obtaining reliable results with a reasonable calculation time. The influence of the turbulence model in terms of comparison presented in this work [38] shows no significant change in the characteristics corresponding to the linear part of the *C_L_* change.

**Figure 12:**
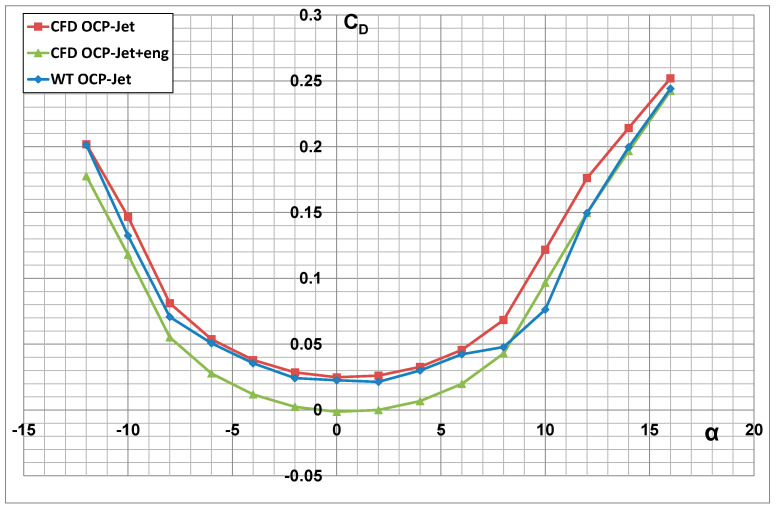
Aerodynamic drag characteristics of an aerial target with the engine thrust effect (CFD OCP-Jet-eng) and without (CFD OCP-Jet).

**Figure 13:**
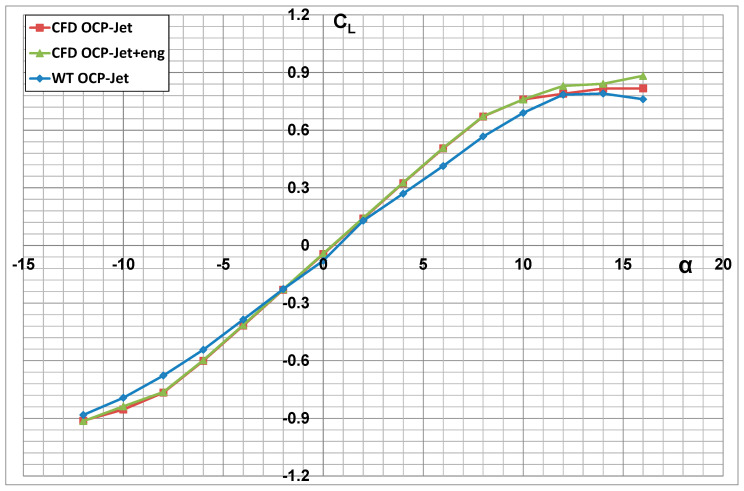
Aerodynamic lift characteristics of an aerial target with engine thrust effect (CFD OCP-Jet-eng) and without (CFD OCP-Jet).

**Figure 14:**
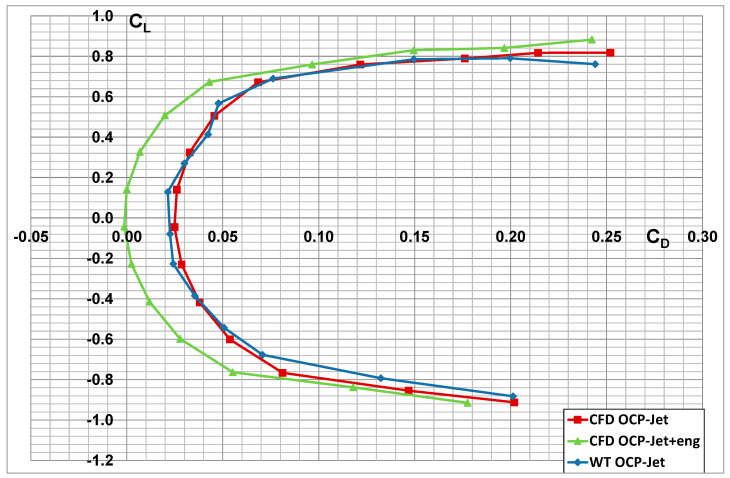
Comparison of *C_L_* = f(C_D_) characteristics of an aerial target with engine thrust effect (CFD OCP-Jet-eng) and without (CFD OCP-Jet).

Numerical analyses were performed in symmetric flow for a flight velocity V = 491 km/h (Mach = 0.4), in the range of angles of attack *α* = −12° ÷ +16°, at sea level altitude, for standard atmospheric parameters: pressure p = 101,325 Pa, temperature T = 288.15 K, and air density ρ = 1.225 kg/m^3^. Figure 12, Figure 13 and Figure 14 present the effect of the engine thrust on the selected aerodynamic characteristics of the aerial target (OCP-Jet aircraft). The characteristics obtained for the case including engine thrust are described as follows for the CFD OCP-Jet + eng. The effect of engine thrust on the value of the drag force and the lift force coefficients is most evident in the polar diagram shown in Figure 14. Furthermore, it can be seen from the characteristics of the drag force coefficient that the effect of the engine thrust on its value becomes less as the angle of attack increases. Furthermore, for this case, C_DMIN_ ≈ C_D0_. The engine thrust in the numerical analysis did not change the slope of the characteristic of the lift force coefficient characteristic ∂*C_L_*/∂*α*.

The results of the numerical analysis were compared with the experimental investigations [38,39] of the “clean” configuration OCP-Jet aircraft model (WT OCP-Jet). Attention is drawn to the high correspondence between the results obtained in the numerical analysis and the results of the experimental tests. This indicates the correctness of the numerical model developed for the OCP-Jet plane for aerodynamic analysis. Possible differences in values of individual aerodynamic coefficients result directly from the specifics of the conducted experimental tests, among others from different values of criterion numbers. The CFD model was tested in 1:1 scale but the wind tunnel model was built in 1:4 scale, so it provides the opportunity to develop a laminar flow separation on the tail surfaces having very short chords, which will be more visible in WTT and influence mostly the moments rather than a force value.

**Figure 15:**
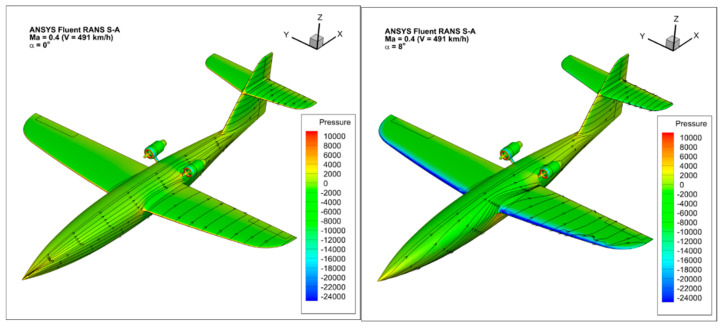
Pressure distribution on the surface of the aircraft airframe for different values of the angle of attack *α*, respectively: 0°, 6°.

In addition, Figure 15 shows a qualitative comparison of the results obtained for selected angles of attack in the form of a pressure map with the path lines shown on the surface of the OCP-JET. Figures show that as the angle of attack increases, the area of vacuum on the upper surface of the wing increases. For smaller angles of attack, the area of negative pressure forms on the leading edge of the wings.

## 4. Results and Discussion

The primary objective of the aerodynamic tests and numerical analysis performed was to obtain reliable information on the aerodynamic properties of the newly designed Jet-propelled Aerial Target. Considering the assumed high maneuverability of this aerial vehicle, it is extremely important to know this type of data during the design process. Based on the results of this work, the following conclusions were pointed out:

- The lowest value of the drag coefficient was obtained for the model with elevator deflection d_H_ = −10° and it was C_D_ = 0.01599;
- The characteristics of the lift force coefficient are linear in the range of angles of attack from *α* = −10° to *α* = 10°;
- The aerial target model is statically stable longitudinally in the range of the AOA from *α* = −16° to *α* = 12°;
- Elevator deflection causes the characteristic *C_m_* = f(*α*) to shift downwards;
- For all the configurations of the tested model, the optimum angle of attack is *α*_opt_ = 8°;
- The maximum value of aerodynamic efficiency was obtained for d_H_ = 0°;
- In an asymmetric flow, the maximum value of the drag force coefficient C_D_ = 0.178 was obtained for both the sideslip angles *β* = −30° and *β* = 30°;
- The aerial target model is statically stable directionally in the investigated range of sideslip angles;
- The effect of engine thrust on the drag force coefficient value becomes less as the AOA increases;
- The thrust of the engine did not change the slope of the characteristic of the lift force coefficient characteristic *∂C_L_*/*∂α*;
- A high and satisfied comparability was found between the results of the numerical analysis and the experimental tests.

The results of the presented work were used to determine the external loads needed for the strength analysis of the composite structure of the airframe.

## 5. Conclusions

Analysis and assessment of aerodynamic properties is a fundamental element of the aircraft design process. In the case of the tested air target, both classic tests of the object model in a wind tunnel and advanced CFD numerical methods were used to assess the aerodynamics. The methods used separately are not perfect and only the use of both at the same time allows for a more accurate assessment of the aerodynamic properties of the object. This combined experimental and numerical research procedure was used for the tested air target. Both methods confirmed that the required performance parameters will be met by the designed aircraft. Moreover, it was confirmed by the aircraft’s flight qualification tests at the Polish Air Force training range [40]. This way, errors and design corrections were avoided, as well as building and testing another prototype. In our case, the first prototype already achieved, and even exceeded, the required performance, clearly reducing the project costs. The experience acquired by the team will be used to design new, both simple and complex, aircraft, in accordance with the requirements of EASA CS 23 and CS 25.

## Figures and Tables

**Table 1:** The impact of elevator deflection on the drag coefficient.

d_H_°	−30°	−20°	−10°	0°	10°	20°	30°
*α*° (C_D min_)	2	2	2	2	0	0	0
C_D min_	0.03162	0.02663	0.01599	0.02136	0.02352	0.03363	0.03528
C_D_ (*α* = −30°)	0.61855	0.60718	0.58583	0.58911	0.58438	0.55594	0.57084
C_D_ (*α* = −10°)	0.1501	0.13894	0.13428	0.13237	0.12347	0.12647	0.11383
C_D_ (*α* = −4°)	0.05926	0.04835	0.04077	0.03544	0.03888	0.04248	0.04689
C_D_ (*α* = 0°)	0.03309	0.02963	0.02746	0.02253	0.02352	0.03363	0.03528
C_D_ (*α* = 4°)	0.03661	0.03732	0.02446	0.02998	0.03376	0.04272	0.04925
C_D_ (*α* = 10°)	0.07361	0.07455	0.07626	0.07619	0.08473	0.08854	0.103
C_D_ (*α* = 30°)	0.50115	0.49621	0.51205	0.52973	0.54183	0.55702	0.56464

**Table 2:** Influence of elevator deflection on lift force coefficient.

d_H_°	−30°	−20°	−10°	0°	10°	20°	30°
*α*° (*C_L_* = 0)	2	1.5	1	1	0.5	0	0.5
*C_L_* (*α* = −10°)	−0.89919	−0.8909	−0.85761	−0.7930	−0.75293	−0.73197	−0.68523
*C_L_* (*α* = 0°)	−0.1699	−0.1425	−0.1178	−0.079	−0.0394	−0.0047	0.02893
*C_L_* (*α* = 10°)	0.57024	0.6099	0.63362	0.68967	0.72973	0.75795	0.79622
*C_L_* _min_	−0.9675	−0.9937	−0.9402	−0.9100	−0.8646	−0.8349	−0.8252
*α*_cr_°	12	12	12	12	12	12	12
*C_L_* _max_	0.8030	0.8101	0.8405	0.8611	0.8734	0.8912	0.90296

**Table 3:** Influence of elevator deflection on pitching moment coefficient.

d_H_°	−30°	−20°	−10°	0°	10°	20°	30°
*α*° (*C_m_* = 0)	26	17	14	2	−11	−12	−15
*C_m_* (*α* = −10°)	0.51132	0.41521	0.33886	0.12631	−0.08181	−0.15985	−0.2618
*C_m_* (*α* = 0°)	0.3866	0.1785	0.1417	−0.0287	−0.1967	−0.2942	−0.3984
*C_m_* (*α* = 10°)	0.21151	0.1502	0.01633	−0.19469	−0.37356	−0.46317	−0.5879
*C_m_* (*α*_kr_)	0.2377	0.1811	0.0165	−0.1790	−0.3436	−0.4462	−0.5051
*C_m_* _min_	−0.1245	−0.0205	−0.1988	−0.2577	−0.3894	−0.5154	−0.5328
*C_m_* _max_	0.7609	0.6702	0.5533	0.4342	0.31089	0.2000	0.3080

**Table 4:** Influence of elevator deflection on aerodynamic efficiency.

d_H_°	−30°	−20°	−10°	0°	10°	20°	30°
−*α*_opt_°	−8	−6	−6	−4	−6	−8	−8
E_min_	−8.437	−10.094	−10.799	−10.880	−10.263	−8.377	−8.337
E (*α* = 0°)	−5.1342	−4.808	−4.291	−3.529	−1.676	−0.141	0.820
*α*_opt_°	8	8	8	8	8	6	8
E_max_	7.794	9.256	10.790	11.865	11.397	10.043	9.207

## Data Availability

The original contributions presented in the study are included in the article, further inquiries can be directed to the corresponding author.
